# Differential Drug Resistance Acquisition in HIV-1 of Subtypes B and C

**DOI:** 10.1371/journal.pone.0000730

**Published:** 2007-08-15

**Authors:** Esmeralda A.J.M. Soares, André F.A. Santos, Thatiana M. Sousa, Eduardo Sprinz, Ana M.B. Martinez, Jussara Silveira, Amilcar Tanuri, Marcelo A. Soares

**Affiliations:** 1 Departamento de Genética, Universidade Federal do Rio de Janeiro, Rio de Janeiro, Rio de Janeiro, Brazil; 2 Hospital de Clínicas de Porto Alegre, Universidade Federal do Rio Grande do Sul, Porto Alegre, Rio Grande do Sul, Brazil; 3 Departamento de Patologia, Fundação Universidade Federal do Rio Grande, Rio Grande, Rio Grande do Sul, Brazil; National Cancer Institute at Frederick, United States of America

## Abstract

**Background:**

Subtype C is the most prevalent HIV-1 subtype in the world, mainly in countries with the highest HIV prevalence. However, few studies have evaluated the impact of antiretroviral therapy on this subtype. In southern Brazil, the first developing country to offer free and universal treatment, subtypes B and C co-circulate with equal prevalence, allowing for an extensive evaluation of this issue.

**Methods and Findings:**

Viral RNA of 160 HIV-1+ patients was extracted, and the protease and reverse transcriptase genes were sequenced, subtyped and analyzed for ARV mutations. Sequences were grouped by subtype, and matched to type (PI, NRTI and NNRTI) and time of ARV exposure. Statistical analyses were performed to compare differences in the frequency of ARV-associated mutations. There were no significant differences in time of treatment between subtypes B and C groups, although they showed distinct proportions of resistant strains at different intervals for two of three ARV classes. For PI, 26% of subtype B strains were resistant, compared to only 8% in subtype C (*p* = 0.0288, Fisher's exact test). For NRTI, 54% of subtype B strains were resistant *versus* 23% of subtype C (*p* = 0.0012). Differences were significant from 4 years of exposure, and remained so until the last time point analyzed. The differences observed between both subtypes were independent of time under rebound viremia in cases of virologic failure and of the number of HAART regimens used by treated patients.

**Conclusions:**

Our results pointed out to a lower rate of accumulation of mutations conferring resistance to ARV in subtype C than in subtype B. These findings are of crucial importance for current initiatives of ARV therapy roll-out in developing countries, where subtype is C prevalent.

## Introduction

The genetic diversity of human immunodeficiency virus type 1 (HIV-1) allows for its classification in several groups, subtypes, sub-subtypes and circulating recombinant forms (CRF) [1]. To date, 9 known subtypes and at least 34 CRF are heterogeneously distributed around the world. While subtype B predominates in developed countries of Western Europe and U.S., other (non-B) subtypes or CRF account for the majority of infections in the developing world [2]. Interestingly, subtype C is responsible for 50% of global HIV infections [2], in countries with the highest known prevalence (in sub-Saharan Africa), and with large populations, like India [3], [4].

An increasing body of experimental evidence suggested that different HIV-1 subtypes might exhibit disparate biological behaviors, and might respond differently to diagnostic, immunologic and therapeutic interventions [5]–[7]. With respect to HIV antiretroviral (ARV) treatment, recent studies identified subtype-specific differences in viral susceptibility to specific drugs [8], [9] and in signature mutations selected by treatment [10]–[12]. An important problem, in this scenario, is whether HIV-1 subtypes may differ in the rate of fixation of mutations conferring drug resistance in individuals under ARV therapy, a point recently addressed in a single report [13].

As Brazil exhibits a heterogeneous HIV-1 subtype distribution [14], [15] and a history of universal and free access to ARV therapy since 1996 [16], it represents an appropriate setting for retrospectively analyzing the rate of fixation of mutations conferring drug resistance under specific ARV class exposure, in different subtypes. In particular, we selected the southernmost state of Brazil, Rio Grande do Sul, as study site because it is characterized by an equal distribution of B and C subtypes [17], [18] co-circulating in individuals under similar socio-demographic conditions. In this report, we found that subtype C is less prone to fix mutations conferring drug resistance over time when compared to subtype B counterparts subjected to the same type and length of ARV drug exposure, in two of three ARV classes.

## Methods

### Patients

Regularly followed patients, at two public health system hospitals in the state of Rio Grande do Sul (Hospital de Clínicas de Porto Alegre and Hospital Universitário do Rio Grande), participated in this study. One hundred and sixty patients were initially enrolled. Eligibility criteria included age above 18 years, current exposure to ARV therapy at time of sampling and availability of all clinical, laboratory (CD4^+^ T-cell counts and HIV viral load) and treatment histories. Viral load measurements were conducted with the Quantiplex HIV-1 RNA 2.0 Assay (Bayer Diagnostics, Tarrytown, NY, U.S.). Only patients with self-reported adherence to therapy during all treatment periods were included in analyses. Following written informed consents (provided by 100% of participants), plasma specimens were collected and available medical records were reviewed. All sample collections were conducted from July 2002 to January 2003. This study was approved by the Internal Review Boards from both Institutions, the *Comitê de Ética em Pesquisa do Hospital de Clínicas de Porto Alegre* and the *Comitê de Ética em Pesquisa na Área de Saúde da Fundação Universidade Federal do Rio Grande*.

### HIV-1 molecular characterization and drug resistance analysis

Viral RNA was extracted from plasma and complementary DNA synthesis was carried out with random primers as previously described [19]. Nested polymerase chain-reactions (PCR) were conducted with specific primers. The entire protease region (PR) and the first 225 codons of reverse transcriptase (RT) were amplified, purified with the Qiagen PCR purification kit (QIAGEN, Valencia, CA) and sequenced in an ABI3100 automated sequencer (Applied Biosystems, Foster City, CA). Sequences were aligned with SeqMan (DNAStar, Madison, WI) and manually edited. Edited sequences were subsequently aligned, with ClustalW [20], to reference sequences representative of all HIV-1 subtypes available at the Los Alamos database (hiv-web.lanl.gov). Aligned sequences were subjected to phylogenetic analyses by neighbor-joining and Kimura 2-parameter model of the MEGA 3.0 package for inference of HIV-1 subtypes [21]. Identification of antiretroviral resistant mutations in PR and RT genes was carried out following electronic submission to the Stanford University database (hivdb.stanford.edu) [22]. Mutations were recorded according to the International AIDS Society-USA consensus [23]. Sequence data were submitted to the GenBank with accession numbers AY275719-AY275807, AY390079-AY390081, AY390178-AY390190, DQ190959-DQ191030, DQ343964-DQ344016, and DQ659454-DQ659487.

### Data groups and statistical analyses

Demographic and clinical data from patients (age, gender, time of HIV diagnosis, CD4 T-cell counts, HIV viral load, CDC immune and clinical staging and treatment status and time of treatment) were compiled for each subtype (B and C) group and differences were analyzed with Student's *t*-test or Fisher's exact test.

Sequences were grouped according to their assigned HIV-1 subtype, and further separated according to type of ARV exposure, nucleoside/nucleotide RT, non-nucleoside RT or protease inhibitors (NRTI, NNRTI and PI, respectively). Finally, viral sequences were further grouped according to time of ARV exposure (in 12-month periods) within each ARV class. Cumulative curves of proportions of mutant viruses within each group over ARV exposure time were plotted for each subtype (B and C). Significant differences in the proportion of mutants at each time point were evaluated by one-tailed Fisher's exact tests, with significance level  = 0.05.

The same analysis was also conducted for patients subjected exclusively to HAART, to avoid confounding factors of drug resistance generation by sub-optimal regimens (mono and/or dual therapy). Additionally, we have compared the proportion of patients subjected to one or more than one HAART regimen, as well those that received mono and/or dual therapy prior to HAART in both subtypes. The average number of mutations per genome, as well as the proportion of resistant strains in those three groups was also compared in both subtypes.

Exposure times to individual antiretrovirals were also compiled for each subtype group for evaluating differences with respect to specific drugs.

We have also assessed the impact of length of rebound viremia in treated patients on the acquisition of drug resistance mutations in both subtypes, by comparing the average time of rebound viremia in both groups through Student's t test. The proportion of resistant viruses among those patients with rebound viremia was also compared through Fisher's exact test. Finally, we have also compared the proportion of patients reaching undetectable viral load after HAART initiation over time in both subtype groups by Fisher's exact test.

## Results

### Demographic, clinical and molecular profiles

One hundred and sixty patients with known ARV treatment history were screened for infecting HIV-1 subtype by RT-PCR, by sequence and phylogenetic analyses of partial *pol* sequences. Of these, 136 were infected with either subtype B (n = 84) or C (n = 52), which were selected for further analysis. The 24 remaining viral isolates corresponded to subtype F1 (n = 11), subtype D (n = 4), and mosaic forms (n = 9).

Table 1 summarizes major demographic and clinical parameters for subtype B- and C-infected groups. Of these parameters, time elapsed from diagnosis to sample collection differed significantly between both groups, being shorter for subtype C-infected patients (*p* = 0.013; Student's t-test). The proportion of patients in each subtype group classified in CDC clinical stage C also differed significantly (*p* = 0.05; Fisher's exact test). Finally, subtype B-infected patients showed lower CD4 T-cell counts than subtype C at HAART initiation (*p* = 0.003; Table 1).

**Table 1:** Demographic and clinical characteristics of HIV-1-positive treated patient groups infected with B and C subtypes.

	SUBTYPE B (n = 84)	SUBTYPE C (n = 52)	*p* value
Age (yr/SD)	40.59 (10.86)	40 (12.51)	0.779
Gender (%)			0.131
Male	52 (62)	29 (58)	
Female	32 (38)	21 (42)	
Average time of diagnosis (yr/SD)	6.42 (3.88)	4.93 (3.02)	**0.013** *
CDC Clinical Stage (%)			
A	23 (27.71)	16 (31.38)	0.139
B	20 (24.10)	17 (33.33)	0.080
C	40 (48.19)	18 (35.29)	**0.050**
CDC Immune Stage (%)			
1	19 (22.62)	11 (21.57)	0.170
2	40 (47.62)	23 (47.06)	0.142
3	25 (29.76)	15 (31.37)	0.154
Average CD4 T-cell counts at HAART initiation (SD)	135 (108)	254 (154)	**0.003**
Average CD4 T-cell counts at sample collection (SD)	334 (212)	334 (195)	0.937
Median log10 viral RNA prior to HAART initiation	5.2	5.0	0.975
Median log10 viral RNA at sample collection	2.6	2.0	0.198
Undetectable VL (%) (<80 copies/ml plasma)	28 (33.7)	22 (44.8)	0.065
Average time of ARV treatment (yr/SD)			
NRTI/Total	3.58 (2.18)	2.97 (2.15)	0.116
NNRTI	1.8 (0.98)	1.49 (0.99)	0.187
PI	2.72 (1.83)	2.59 (1.4)	0.687

* Bold *p* values are significant at the 0.05 level

All amino acid-deduced *pol* sequences from treated patients were analyzed for drug resistance mutations according the IAS-USA consensus [23]. Most of the RTI- and PI-associated mutations occurred in both subtype groups, suggesting that qualitative patterns of drug resistance acquisition were similar. Some RTI (K65R, L74V) and PI (D30N, M46I/L, I84V) mutations were only seen in subtype B isolates. Conversely, one multi-NRTI resistant genotype (A62V, V75I, F116Y and Q151M) was found among subtype C isolates. Of note, the thymidine analogue mutations M41L, L210W and the 3TC-associated mutation M184V/I, as well as the PI mutation L90M, were significantly more frequent in subtype B isolates (*p*<0.05, data not shown). Interestingly, when both patient groups were analyzed with respect to the number of average mutations per viral genome, subtype B showed higher numbers for all ARV classes, but only the difference for protease inhibitors was found significant (0.6 mutations per genome in subtype B *versus* 0.12 in subtype C; *p*<0.01, Student's *t* test). This higher accumulation of mutations in subtype B isolates could not be explained by longer exposure to ARV therapy because it did not differ from subtype C exposure, either *per* class or globally (Table 1). Despite similar times of total ARV exposure, subtype B might have been exposed to mono and dual therapy for a longer time because this subtype has been circulating in Brazil for a longer period than subtype C, at a time when these regimens were common. This could have selected mutations faster in subtype B than in C. However, analysis of previous time of exposure to mono and/or dual therapy in both groups failed to show significant differences (*p* = 0.9629, Student́s *t* test, data not shown).

**Figure 1:**
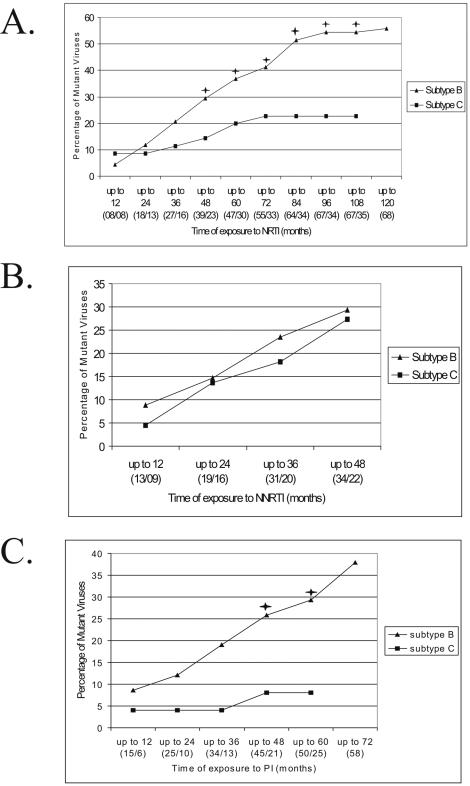
HIV-1 subtype B and C viruses with at least one primary resistance mutation. Different treatment exposure periods (in months) were plotted for each subtype and for each ARV drug class. (A) NRTI, (B) NNRTI, (C) PI. Black asterisks denote significance in the difference of proportions between subtypes B and C at the 0.05 *p* value level.

We further examined the lower accumulation of drug resistance mutations in subtype C isolates compared to subtype B counterparts by analyzing mutations in individual ARV classes. For PI, 38% of subtype B isolates presented at least one primary resistance mutation *versus* only 8% of subtype C isolates (*p* = 0.0037, Fisher's exact test). For NRTI, 56% of subtype B isolates presented primary resistance *versus* 23% of subtype C isolates (*p* = 0.0009, Fisher's exact test). For NNRTI, no differences in the proportion of resistant isolates were found between subtypes. In addition to the ARV class exposure, all patients in both groups had their complete treatment history assessed and were stratified according to time of exposure to each ARV class. Patients were cumulatively pooled in increasing time periods, and the percentage of strains carrying primary mutations was compared in both subtype groups (Figure 1 shows these comparisons for each ARV class). For NRTI-related mutations, subtype C viruses differed significantly from subtype B from 4 year-exposure, and this difference steadily increased to 9 year-exposure (Figure 1A). At this point, 54% of subtype B viruses harbored NRTI-related mutations, whereas less than half (23%) of subtype C viruses carried these mutations (*p* = 0.0012, Fisher's exact test). Protease resistance mutations occurred significantly at higher levels in subtype B already by 4 years of PI exposure, and also remained significantly higher for up to 5 years (26% in subtype B *versus* 8% in subtype C; *p* = 0.0288, Fisher's exact test; Figure 1C). In agreement with previous analyses, NNRTI mutations did not differ significantly in both subtype groups, at least to 4 years of exposure, the longest available period (Figure 1B).

**Figure 2:**
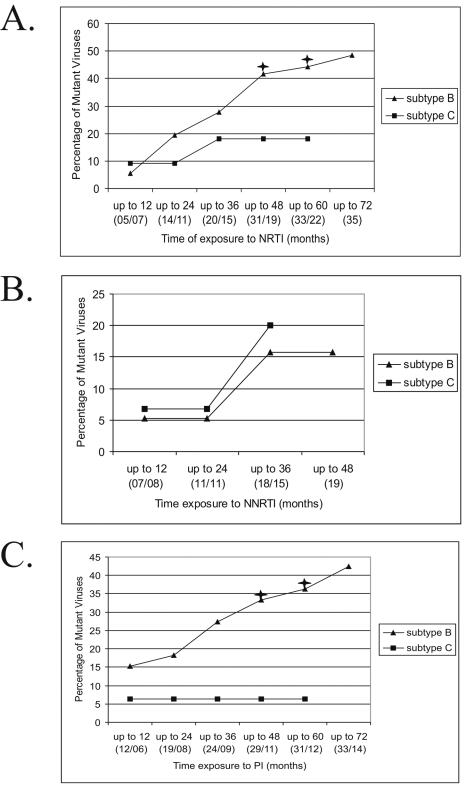
HIV-1 subtype B and C viruses with primary resistance mutations from individuals undergoing HAART. Different treatment exposure periods (in months) were plotted for each subtype and for each ARV drug class. (A) NRTI, (B) NNRTI, (C) PI. Black asterisks denote significance in the difference of proportions between subtypes B and C at the 0.05 *p* value level.

Despite that subtype B and C-infected groups did not differ significantly in time of mono and/or dual therapy previous to HAART, we further restricted our analyses to patients exclusively subjected to HAART (Figure 2). The acquisition of resistance mutations for all ARV classes was identical to those found in global analyses, indicating that NRTI- and PI-associated mutations accumulated faster for subtype B from year 4 of exposure, while NNRTI mutations did not differ between both groups.

It was possible that the number of resistance mutations was associated with a differential use of sequential HAART regimens. To evaluate this, we have compared patients with one or multiple HAART regimens in each subtype group with respect to the average number of mutations per genome and the proportion of resistant strains. Despite the fact that similar proportions of patients were subjected to multiple regimens in both groups (52% of subtype B versus 42% of subtype C, p = 0.11), subtype C had lower proportion of resistant strains and lower average number of mutations in all compared treatment groups (Table 2). Even in patients previously subjected to mono and/or dual therapy, the number of resistant strains was significantly higher for subtype B (Table 2).

**Table 2:** Comparison of drug resistance mutation acquisition between subtype B- and C-infected patients under different therapeutic regimens.

	Subtype B	Subtype C	p-value
One HAART Regimen			
Proportion of isolates with at least one primary resistance mutation	33% _(8/24)_ a	9.5% _(2/21)_	**0.048** b
Average number of drug resistance mutations per isolate _(SD)_	0.5 _(0.78)_	0.1 _(0.3)_	**0.025** c
Multiple HAART Regimens			
Proportion of isolates with at least one primary resistance mutation	62% _(16/26)_	20% _(03/15)_	**0.001**
Average number of drug resistance mutations per isolate _(SD)_	1.8 _(3)_	0.4 _(0.8)_	**0.027**
Previous mono- and/or dual therapy			
Proportion of isolates with at least one primary resistance mutation	56% _(15/27)_	25% _(04/16)_	**0.039**
Average number of drug resistance mutations per isolate _(SD)_	2.4 _(2.9)_	1.1 _(2.4)_	0.130

Statistically significant p-values (<0.05) are in boldface

a Number of patients with resistance/total

b Fisher's exact test

c Student's t test

We have also evaluated the role of each individual ARV drug in the appearance of drug-associated mutations. We calculated the time of exposure to individual ARV in both subtype groups, taking into account the global patient dataset, as well as for those only subjected to HAART (Table 3). Differences in time of exposure to D4T, 3TC and NFV were seen in the global dataset, but these differences only remained significant for NFV when analyzing patients exclusively subjected to HAART. This demonstrated that in these patients, without significant differences in exposure to D4T and 3TC, there was a higher accumulation of NRTI-associated mutations in subtype B (Figure 2A).

**Table 3:** Mean time of drug exposure in months for each HIV-1 subtype-infected group.

Drug£	Subtype B	Subtype C	T-test
	Total/HAART#	Total/HAART	Total/HAART
AZT	27.91/20.98	28.36/19.27	0.9043/0.6327
DDI	24.24/18.58	22.57/16.83	0.7206/0.4501
D4T	27.3/27.6	19/20.1	**0.0218** * **/**0.1375
3TC	27.6/24.4	21.4/19.5	**0.0304**/0.1086
DDC	15.17/17.25	20.75/29.67	0.3494/0.3236
NFV	24.52/23.33	15.61/11.4	**0.0092/0.0052**
RTV	19.03/15.69	15.32/14.45	0.3227/0.7425
IDV	19.71/14.82	22.4/24.58	0.5349/0.1080
SQV	20.29/16.17	18.44/23.33	0.6961/0.3945
LPV	8.44/5.75	7.38/7.8	0.6887/0.8749
NVP	18.88/20	22.11/23.4	0.6515/0.7590
EFV	18.41/18.45	13.79/12.52	0.0781/0.0662

£ The antiretroviral drugs tenofovir and abacavir were not available to patients in the Public Health System at the time of survey

# Values left to the bar correspond to the total treated patients analyzed in the study; those at right represent patients which were only subjected to HAART therapy

* Comparative *T*-test values in bold are those significant at the 0.05 level

**Figure 3:**
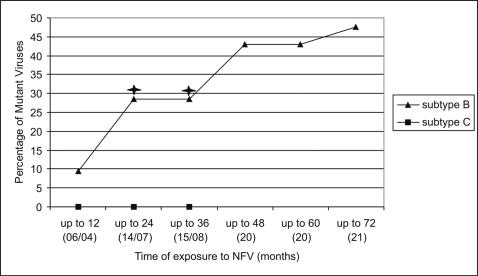
HIV-1 subtype B and C viruses with protease resistance mutations from individuals undergoing NFV-based HAART. Different treatment exposure periods (in months) for that drug were plotted for each subtype. Black asterisks denote significance in the difference of proportions between subtypes B and C at the 0.05 *p* value level.

Since we observed a longer NFV exposure in subtype B patients, this ARV might explain the differences observed between subtype groups in the PI class (Figures 1C and 2C). In fact, half (10/21) of subtype B-infected patients under NFV presented NFV-associated mutations, while none of the subtype C isolates (0/8), under NFV exposure, showed these mutations. For this reason, we analyzed PI-associated mutations in all patients under NFV as PI component of their HAART regimen. This showed that subtype B still accumulated more NFV-associated mutations (primary or secondary) even when matching time of exposure between subtype groups (Figure 3). Around 12 months of NFV exposure, some 10% of subtype B strains already carried NFV-associated mutations, while none of subtype C strains was resistant to NFV. By 24 and 36 months, approximately 30% of subtype B strains already carried NFV mutations, and differences in the proportion of resistant strains was already statistically significant (*p* = 0.05; Figure 3). Thus, despite NFV had been used more extensively in subtype B-infected subjects, it resistance mutations were positively selected more rapidly when matching exposure times were analyzed.

We have also investigated the impact of rebound viremia time on the accumulation of drug resistance. It was plausible that subtype B patients could have accumulated more mutations because they have had failed therapy for longer than those infected with subtype C. We found 24 subtype B- and 17 subtype C-infected patients with rebound viremia. We have calculated the average time of rebound viremia for each subtype group, and found that they do not differ significantly (15.6 months for subtype B and 19 months for subtype C). Interestingly, we found that the proportion of those patients carrying drug resistant viruses was significantly higher for subtype B (46%) than for subtype C (18%) (p = 0.048, Fisher's exact test). We have also checked the proportion of subtype B- and C-infected patients who had undetectable VLs over time of therapy, and they did not differ significantly at any time point, except for the initial suppression period (until 8 months after HAART initiation). At that time, the subtype C group had 79% of patients with undetectable VL, compared to only 52% of the subtype B group (p = 0.04, Fisher's exact test).

## Discussion

The impact of ARV treatment on different HIV-1 subtypes and CRF is an issue of paramount concern associated to the introduction of treatment in developing countries. To date, however, scarce data are available on the impact of ARV in HIV-1 of non-B subtypes, which paradoxically account for almost 90% of all HIV infections in the world. Southern Brazil represents an ideal setting for assessing this question because subtypes B (the most studied) and C (the most prevalent) co-circulate in very similar frequencies in the same population [15], [17], [18].

Our group has previously shown that subtype C was introduced in Brazil later than subtype B [15], [18], [24], in agreement with our observation of a shorter time of diagnosis for individuals infected with subtype C (Table 1). Although the HIV subtype B epidemic is older, the time of treatment did not differ significantly between B and C subtypes for all drug classes (Table 1).

When analyzing the rate of accumulation of mutations conferring drug resistance over time for each major ARV class, subtype C viruses apparently acquired a lower number of mutations than subtype B for PI and NRTI, but not for NNRTI. We ruled out the possibility that previous exposure to mono- or dual-therapy in subtype B-infected patients (affected by an older epidemic) might explain these differences. To further investigate the rate of accumulation of mutations, we stratified subtype groups by time of therapy at 12 month-intervals. Here again, different rates between subtypes B and C were observed, for PI and NRTI mutations, but not for NNRTI. Our data were further confirmed by similar analyses in patients exclusively subjected to HAART. In this setting, we definitively excluded the possibility that previous mono and/or dual therapy accounted for these differences. Of note, HAART was universally initiated in Brazil with the introduction of PI in 1996, when the prevalence of subtypes B and C were already very similar [17], [18].

We ruled out that time of exposure to individual ARV drugs, rather than ARV classes, accounted for different mutation rates between subtypes B and C. In fact, analysis of the global dataset showed that D4T, 3TC and NFV were more extensively used in subtype B patients (Table 3) but the significance of D4T and 3TC exposure disappeared when analysis was restricted to HAART patients. This provided strong evidence that differences in NRTI-related mutations, still present in HAART patients, could not be attributed to a differential exposure to these drugs. As the use of NFV was significantly higher in subtype B isolates, we carried out separate analysis of the time of exposure to this drug matching exposure time. This analysis showed that subtype B accumulated NFV-related mutations more rapidly than subtype C.

We have also ruled out the impact of treatment failure time on the increased drug resistance accumulation in subtype B. Both subtypes had similar rebound viremia times, but subtype B retained a higher proportion of resistant strains in failed individuals. The proportion of virological success (undetectable viral load, uVL) between subtype groups was similar in all time periods after HAART initiation, with the exception of the period spanning 5–8 months of therapy, where a higher proportion of subtype C had more patients with uVL.

The use of distinct drug regimens (single or multiple HAART, or previous use of mono and/or dual therapy) did not seem to influence the higher rates of drug resistance acquisition in subtype B compared to C. For all types of ARV exposure, the proportion of resistant strains and the average number of mutations per genome was lower in subtype C-infected patients, with exception of patients subjected to mono/dual therapy.

Our observations were unexpected and, to some extent paradoxical, since all ARV drugs were designed for subtype B, which predominates in developed countries [2]. Furthermore, the presence of polymorphisms in non-B subtypes, which are considered as secondary resistance mutations for subtype B [25]–[27], supports the proposition that the acquisition of resistance might be enhanced in non-B subtypes. Holguin *et al*. [28] showed that these polymorphisms do not alter susceptibility of non-B subtypes to ARV drugs, while other authors have reported such differences, pointing out to the increased susceptibility of some non-B subtypes to some ARV [29], [30]. Genotypic analyses of subtype B- and C-infected patients undergoing HAART failure in Israel showed several drug resistance mutations with higher frequency in subtype B viruses [31]. This study supported the hypothesis that subtype C accumulates resistance mutations at lower levels than subtype B.

In our study, NNRTI was the only ARV class for which subtype C did not differ from subtype B with respect to the rate of acquisition of drug resistance mutations. Evidence that NNRTI-related mutations appeared at a higher rate in subtype C was previously reported [13], in agreement with our results.

A straightforward explanation for the findings herein reported can not be provided in view of the complexity of viral biology. Few studies are available on differences between group M subtypes in response to ARV. Recent studies postulated that different subtype-specific genetic barriers might be operating in the development of drug resistance [10], [32]. Accordingly, some subtypes might accumulate resistance at different rates than subtype B at definite amino acid positions in PR and RT. Approximately 30% of subtype C presented a higher genetic barrier for acquiring mutation L210W *versus* 11% among subtype B viruses [32], although differences at this position, *per se*, cannot explain the observed differences for NRTI. It is also conceivable that subtype C might accumulate fewer mutations because is more susceptible to the ARV drugs currently in use, as suggested for other non-B subtypes [29], [30]. In fact, Gonzales *et al*. [7] showed that subtype C strains from Brazil and Africa were naturally hypersusceptible to the PI lopinavir. Moreover, subtypes may be operating under different evolutionary pressures when acquiring specific drug resistance mutations, as demonstrated for the D30N NFV-associated mutation in subtype C [11]. We cannot completely rule out the possibility that we have neglected potentially new, subtype C-specific drug resistance mutations in our analyses. A larger study comparing subtype C-infected drug-naïve and experienced patients identified three new putative positions (two in PR and one in RT) [26]. However, the phenotypic and clinical impact of changes at these codons is yet to be determined. So far, only mutation 89I/V was found associated with PI treatment for subtypes C, F and G [19], but this was stated as a secondary mutation since it does not confer resistance *per se*. Alternatively, we may speculate that intrinsic fitness and replicative capacity of subtypes might account for their ability of accumulating mutations in the infected host. In this respect, it has been shown that subtype C is the least fit of all HIV-1 group M subtypes in competition assays carried out in peripheral blood mononuclear cells, independently of viral tropism [33], [34].

Despite the fact that the lower accumulation of drug resistance mutations in HIV-1 subtype C has not been elucidated, our observations are highly relevant for international treatment roll-out initiatives aiming to extend ARV protocols to developing countries. These are particularly valid for countries of sub-Saharan Africa, which concentrate most of the HIV-infected individuals in the world, mainly by subtype C. Our data may be useful for an aggressive initiation and expansion of ARV therapy in the developing world.
